# Expression of Thymidylate Synthase in Cancer: A Tissue Microarray Study Involving 17,371 Cancers from 136 Tumor Entities

**DOI:** 10.3390/biomedicines14071599

**Published:** 2026-07-16

**Authors:** Florian Lutz, Lisa Sophie Hannemann, Seyma Büyücek, Katharina Möller, Florian Viehweger, Ria Schlichter, Andreas M. Luebke, Martina Kluth, Claudia Hube-Magg, Andrea Hinsch, Christian Bernreuther, Guido Sauter, David Dum, Andreas H. Marx, Ronald Simon, Till Krech, Till S. Clauditz, Frank Jacobsen, Eike Burandt, Stefan Steurer, Patrick Lebok, Christoph Fraune, Sarah Minner, Natalia Gorbokon, Maximilian Lennartz

**Affiliations:** 1 Institute of Pathology, University Medical Center Hamburg-Eppendorf, 20246 Hamburg, Germany; f.lutz@uke.de (F.L.); hannemann.lisa@t-online.de (L.S.H.); s.bueyuecek@uke.de (S.B.); ka.moeller@uke.de (K.M.); f.viehweger@uke.de (F.V.); r.schlichter@uke.de (R.S.); luebke@uke.de (A.M.L.); m.kluth@uke.de (M.K.); a.hinsch@uke.de (A.H.); cbernreuther@uke.de (C.B.); g.sauter@uke.de (G.S.); d.dum@uke.de (D.D.); t.krech@uke.de (T.K.); t.clauditz@uke.de (T.S.C.); f.jacobsen@uke.de (F.J.); e.burandt@uke.de (E.B.); s.steurer@uke.de (S.S.); p.lebok@uke.de (P.L.); c.fraune@uke.de (C.F.); s.minner@uke.de (S.M.); n.gorbokon@uke.de (N.G.); m.lennartz@uke.de (M.L.); 2 Department of Pathology, Academic Hospital Fuerth, 90766 Fuerth, Germany; andreas.marx@klinikum-fuerth.de; 3 Institute of Pathology, Clinical Center Osnabrueck, 49076 Osnabrueck, Germany

**Keywords:** thymidylate synthase, TYMS, immunohistochemistry, tissue microarray, multitumor analysis, tumor phenotype

## Abstract

**Background/Objectives**: Thymidylate synthase (TYMS) represents an important therapeutic target. **Methods**: In this study, TYMS expression was analyzed by immunohistochemistry on a tissue microarray containing 17,371 samples from 136 different tumor types. **Results**: TYMS staining was seen in 42.9% of 15,361 analyzable tumors, with weak staining in 35.4%, moderate in 5.7%, and strong in 1.8%. TYMS occurred in at least one case of 127 categories, of which 71 showed TYMS staining in at least 50% of cases, and 56 included at least one case with strong positivity. TYMS positivity occurred most commonly in lymphomas (81.3–96.5%), sarcomas and sarcomatoid carcinomas (33.3–100%), malignant melanoma (70.5–90.7%), cervical adenocarcinoma (78.3%), and squamous cell carcinomas of various sites (57.1–77.9%). High TYMS expression was linked to advanced pT (*p* = 0.0097), high grade (*p* < 0.0001), ER negativity (*p* < 0.0001), and PR negativity (*p* = 0.0002) in invasive breast cancer of no special type; high grade (*p* < 0.0050), high UICC stage (*p* = 0.0060), and nodal metastasis (*p* = 0.0120) in clear cell renal cell carcinoma (RCC); high grade (*p* < 0.05) and nodal metastasis (*p* = 0.0045) in papillary RCC; high Gleason grade (*p* < 0.0001) and advanced pT stage (*p* = 0.0149) in prostatic adenocarcinoma; high pT (*p* < 0.0001), nodal metastasis (*p* = 0.005), lymphatic (*p* = 0.0064) and venous invasion (*p* = 0.0005), left side location (*p* < 0.0001), and microsatellite instability (*p* < 0.0001) in colorectal adenocarcinoma; and high grade (*p* < 0.0001) in squamous cell carcinomas of different sites. **Conclusions**: TYMS is often overexpressed across different cancer entities and shows associations with several adverse histopathological parameters commonly used to describe tumor phenotypes.

## 1. Introduction

The thymidylate synthase (TS, TYMS), coded by the TYMS gene at chromosome 18p11.32, plays a critical role in the production of thymine, an essential component of DNA. TYMS is expressed in the cytoplasm and nucleus of non-neoplastic and neoplastic cells, where it has an essential function in DNA replication and DNA repair [1,2,3]. The only pathway for the de novo synthesis of thymidine depends on TYMS because TYMS is needed to convert deoxyuridine monophosphate (dUMP) into deoxythymidine monophosphate (dTMP), which is eventually integrated into the DNA [4,5,6,7]. It has been shown that cell types with higher proliferation rates, such as stimulated liver cells after partial hepatectomy [8,9], exhibit higher TYMS expression and activity than cell types with lower proliferation rates [10], and the inhibition of TYMS leads to decreased cell proliferation and cell growth of non-neoplastic and neoplastic cells [1,11].

Various studies have highlighted the important role of the TYMS protein in cancer. Functional studies have demonstrated that TYMS upregulation can be sufficient to induce a malignant phenotype by inducing cell proliferation, cell invasion, and epithelial–mesenchymal transition and reducing apoptosis in cells from hepatocellular carcinoma, pancreatic adenocarcinoma, esophageal squamous cell carcinoma, and colorectal adenocarcinoma [12,13,14,15]. TYMS is the target for several chemotherapeutic drugs, including 5-fluorouracil (5-FU) and methotrexate [16,17]. Some studies have suggested that low TYMS expression may be linked to a better response to these therapies [3,18,19,20]. High levels of TYMS expression have been found to be associated with an aggressive tumor phenotype and poor prognosis in breast cancer [3,21], hepatocellular carcinoma [22], lung cancer [7,23], prostate cancer [24,25] and retroperitoneal liposarcoma [26]. However, data on TYMS protein expressions in cancer are incomplete. RNA expression data are of limited utility because TYMS is always expressed in tumor-associated stromal cells. Immunohistochemistry (IHC) enables a selective analysis of tumor cells, but IHC data on TYMS expression in cancer are not available for all tumor entities and are often discrepant for tumor types that were analyzed by multiple groups. For example, the range of reported TYMS-positive cases ranged from 20.0% to 100% in colorectal adenocarcinoma [27,28], 20.0% to 100% in pulmonary adenocarcinoma [29,30], 21.0% to 100% in invasive breast carcinoma of no special type (NST) [18,31], 7.7% to 97.1% in gastric adenocarcinoma [32,33], 9.6% to 85.3% in pulmonary neuroendocrine tumors [34,35], and 31.2% to 100% in pulmonary squamous cell carcinoma [36,37]. Such variability in data commonly occurs in IHC studies because of the use of different antibodies, staining protocols, and interpretation criteria [38].

To better understand the prevalence and usability of TYMS as a molecular marker in cancer, an extensive and highly standardized survey of TYMS protein expression in a broad range of different tumor types is needed. Given the frequent expression of TYMS in both tumor and stromal cells, IHC represents the only approach that enables a cell-specific assessment of TYMS protein expression within tumor cells. TYMS expression was thus evaluated in more than 17,000 tumor tissue samples from 136 different tumor types and subtypes as well as 76 different non-neoplastic tissue types by IHC in a tissue microarray (TMA) format in this study.

## 2. Materials and Methods

**Tissue Microarrays (TMAs).** Our normal TMA was composed of 8 samples from 8 different donors for each of the 76 different normal tissue types (608 samples on one slide). The cancer TMAs contained a total of 17,371 primary tumors from 136 tumor types and subtypes. Detailed histopathological and molecular data were available for cancers of the breast (*n* = 2075), endometrium (*n* = 259), ovary (*n* = 524), colorectum (*n* = 2351), and kidney (*n* = 2229). The compositions of both normal and cancer TMAs are described in detail in the Section 3. All samples were from the archives of the Institutes of Pathology, University Hospital of Hamburg, Germany; the Institute of Pathology, Clinical Center Osnabrueck, Germany; and the Department of Pathology, Academic Hospital Fuerth, Germany. Tissues were fixed in 4% buffered formalin and then embedded in paraffin. TMA tissue spot diameter was 0.6 mm. The use of archived remnants of diagnostic tissues for manufacturing TMAs and their analysis for research purposes as well as patient data analysis has been approved by local laws (HmbKHG, §12) and by the local ethics committee (Ethics commission Hamburg, WF-049/09). All work has been carried out in compliance with the Helsinki Declaration.

**Immunohistochemistry.** Freshly cut TMA sections were immunostained in one experiment on one day. Slides were deparaffinized with xylol, rehydrated through a graded alcohol series, and exposed to heat-induced antigen retrieval for 5 min in an autoclave at 121 °C in pH 7.8 Tris-EDTA-Citrat (TEC) buffer. Endogenous peroxidase activity was blocked with a Dako REAL Peroxidase-Blocking Solution (Agilent Technologies, Santa Clara, CA, USA; #S2023) for 10 min. A primary antibody specific to TYMS (recombinant rabbit monoclonal antibody (clone-ID HMV305, ardoci, Hamburg, Germany; AO6504-305) was applied at 37 °C for 60 min at a dilution of 1:150. For the purpose of antibody validation, the normal TMA was also analyzed by the mouse monoclonal antibody TYMS/1884 (ab224649, Abcam, Cambridge, UK) at a dilution of 1:100 and an otherwise identical protocol. The bound antibody was then visualized using the Dako REAL EnVision Detection System Peroxidase/DAB+, Rabbit/Mouse kit (Agilent Technologies, Santa Clara, CA, USA; #K5007), according to the manufacturer’s directions. The sections were counterstained with hemalaun. IHC staining was evaluated using a well-established scoring approach previously applied in more than 300 studies from our group (PubMed, May 2026). For tumor tissues, the percentage of positive neoplastic cells was estimated, and the staining intensity was semi-quantitatively recorded (0, 1+, 2+, 3+). For statistical analyses, the staining results were categorized into four groups. Tumors without any staining were considered negative. Tumors with 1+ staining intensity in ≤70% of tumor cells or 2+ intensity in ≤30% of tumor cells were considered weakly positive. Tumors with 1+ staining intensity in >70% of tumor cells, 2+ intensity in 31–70%, or 3+ intensity in ≤30% of tumor cells were considered moderately positive. Tumors with 2+ intensity in >70% or 3+ intensity in >30% of tumor cells were considered strongly positive.

**Statistics.** Statistical calculations were performed with JMP17^®^ software (SAS^®^, Cary, NC, USA). Contingency tables and the chi^2^-test were performed to search for associations between TYMS immunostaining and tumor phenotype.

## 3. Results

### 3.1. Technical Issues

A total of 15,361 (88.4%) of 17,371 tumor samples were interpretable in our TMA analysis. Non-interpretable samples demonstrated a lack of unequivocal tumor cells or a complete lack of tissue. A sufficient number of samples (≥4) of each normal tissue type was evaluable.

### 3.2. TYMS in Normal Tissues

**Figure 1:**
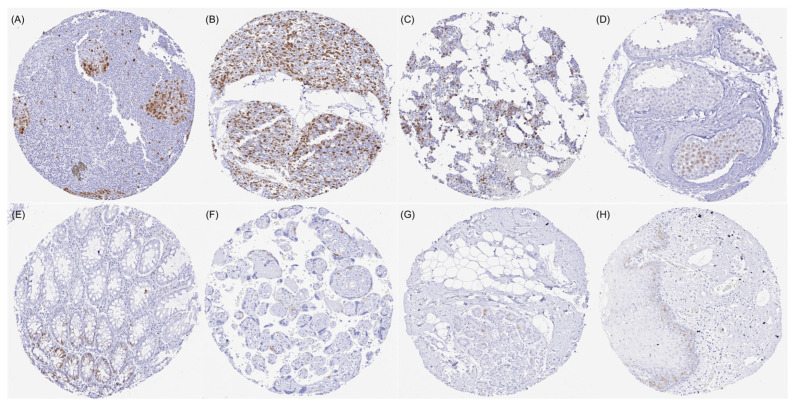
TYMS staining in normal tissues. The panels show strong TYMS staining in many lymphocytic cells of germinal centers and only few interfollicular cells of lymph node (**A**); intense TYMS staining of many lymphocytes of the thymus (**B**); moderate-to-strong TYMS staining of a subset of hematopoietic cells in bone marrow (**C**); moderate TYMS staining in spermatocytes of testis (**D**); weak-to-moderate TYMS staining of a subset of crypt epithelial cells of rectum (**E**); weak-to-moderate TYMS staining of scattered cytotrophoblast cells of the mature placenta (**F**); weak-to-moderate TYMS staining of a subset of luminal epithelial cells of breast glands (**G**); and a weak-to-moderate TYMS positivity of a subset of suprabasal squamous epithelial cells (**H**).

TYMS immunostaining was typically both cytoplasmic and nuclear. It was only detectable in a minority of normal tissues and was often only weak. The strongest TYMS positivity occurred in a subset of lymphoid cells, especially in germinal centers and in the thymic cortex, as well as in a subset of hematopoietic cells in the bone marrow. A usually weak-to-moderate TYMS positivity also occurred in spermatocytes of the testis and in subsets of crypt cells of the colorectum, neck cells of the stomach epithelium, gallbladder epithelial cells, suprabasal cells of non-keratinizing squamous epithelium, luminal glandular cells of the breast, cytotrophoblast, stromal and endothelial cells of the placenta, and endothelial cells in the corpus luteum of the ovary. All of these findings were obtained by using the recombinant rabbit monoclonal antibody HMV305 and the mouse monoclonal antibody TYMS/1884 and therefore were considered to be specific. When using HMV305, TYMS staining was not seen in skin, Brunner glands, salivary glands, endocervical glands, endometrium, fallopian tube, chorion cells, amnion cells, respiratory epithelium, lung, kidney, urothelium, prostate, seminal vesicles, epididymis, thyroid, parathyroid, hypophysis, and the brain. Representative images of normal tissues are shown in Figure 1. By using TYMS/1884, an additional strong cytoplasmic staining was seen in the prostate. This was considered an antibody-specific cross-reactivity of TYMS/1884 (Supplementary Figure S1).

### 3.3. TYMS in Cancer

**Figure 2:**
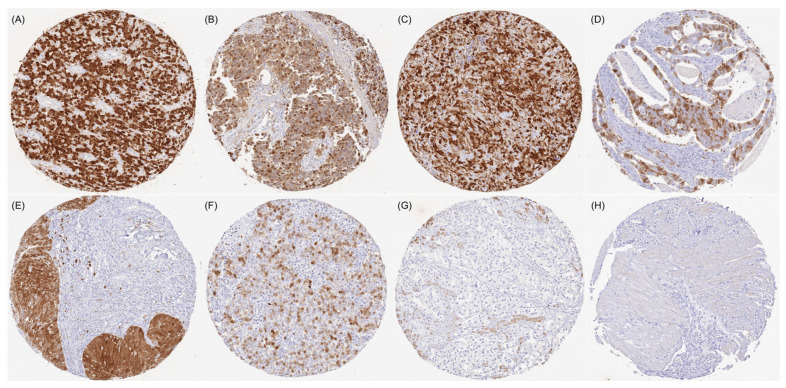
TYMS immunostaining in cancer. TYMS staining was nuclear and cytoplasmic. The panels show a distinct TYMS positivity of a high fraction of tumor cells in cases of diffuse large B cell lymphoma (**A**), rhabdomyosarcoma (**B**), melanoma (**C**), adenocarcinoma of the uterine cervix (**D**), squamous cell carcinoma of the penis (**E**), and testicular seminoma (**F**), while tumor cells lacked TYMS staining in a prostatic adenocarcinoma (**G**) and a neuroendocrine tumor of the ileum (**H**).

**Figure 3:**
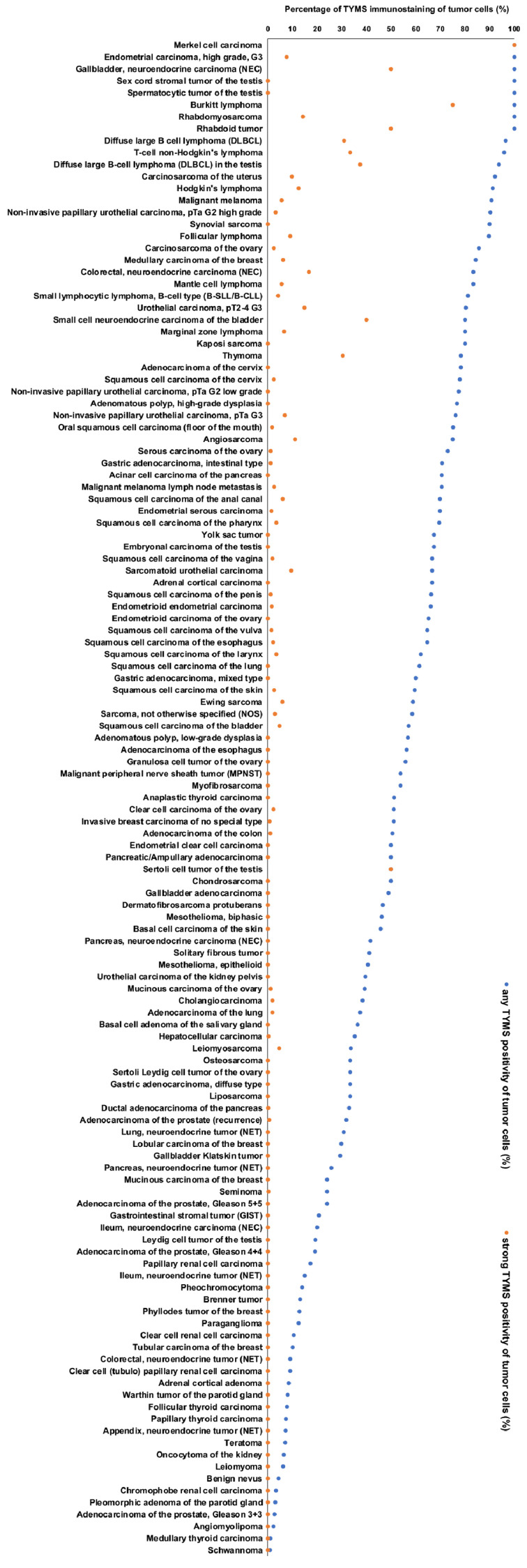
Ranking order of TYMS immunostaining in tumors. Both the percentage of positive cases (blue dots) and the percentage of strongly positive cases (orange dots) are shown.

TYMS staining was seen in 6590 (42.9%) of the 15,361 analyzable tumors. It was considered weak in 35.4%, moderate in 5.7%, and strong in 1.8% of cases. Of 136 tumor categories, 127 showed TYMS expression in at least one case, 71 showed TYMS staining in at least 50% of cases, and 56 included at least one sample with strong TYMS positivity (Table 1). Highest rates of TYMS positivity occurred in B- and T-cell lymphomas (81.3–96.5%), sarcomas and sarcomatoid carcinomas (33.3–100%), primary and metastatic malignant melanoma (70.5–90.7%), adenocarcinoma of the cervix (78.3%), squamous cell carcinomas of various sites (57.1–77.9%), and testicular germ cell tumors (19.4–67.4%). Representative images of TYMS-positive tumors are shown in Figure 2. A graphical representation of the ranking order of TYMS-positive cancers and strongly positive cancers is given in Figure 3. The relationship between TYMS staining and histopathological or molecular features in various cancer types is shown in Table 2. High TYMS expression was linked to advanced pT stage (*p* = 0.0097), high grade (*p* < 0.0001), ER negativity (*p* < 0.0001), PR negativity (*p* = 0.0002), and triple-negative receptor status (*p* < 0.0001) in invasive breast cancer NST; high grade (*p* < 0.005), high UICC stage (*p* = 0.0060), and lymph node metastasis (*p* = 0.0120) in clear cell renal cell carcinoma (RCC); high grade (*p* < 0.05) and lymph node metastasis (*p* = 0.0045) in papillary RCC; high Gleason grade (*p* < 0.0001) and advanced pT stage (*p* = 0.0149) in prostatic adenocarcinoma; advanced pT stage (*p* < 0.0001), lymph node metastasis (*p* = 0.0050), lymphatic (*p* = 0.0064) and venous invasion (*p* = 0.0005); left side (*p* < 0.0001), microsatellite instability (*p* < 0.0001) and BRAF mutations (*p* < 0.0001) in colorectal adenocarcinoma; and high grade in squamous cell carcinomas of different sites (*p* < 0.0001). TYMS expression was not significantly associated with parameters of aggressive disease in endometrial, and ovarian cancer (Supplementary Table S1).

## 4. Discussion

The data from this study provide a comprehensive overview of TYMS protein expression in non-neoplastic and neoplastic human tissues. In line with the essential role of TYMS in DNA replication and DNA repair, our findings show a striking predominance of TYMS immunostaining in tissues with elevated proliferative activity.

**Figure 4:**
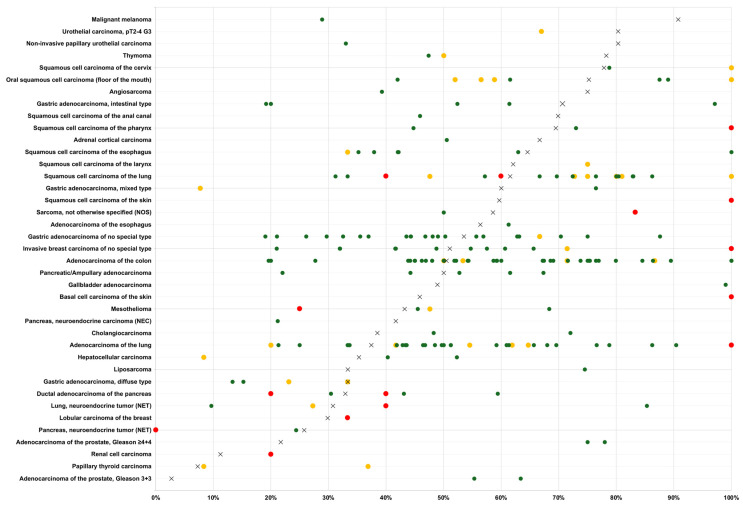
Comparison with the previous TYMS literature. Dots indicate the reported frequencies from the literature for comparison: red dots mark studies with 1–10 analyzed tumors, yellow dots mark studies with 11–25 analyzed tumors, and green dots mark studies with >25 analyzed tumors. Crosses mark our own data.

Given the essential role of TYMS in cells and the ubiquitous expression of the protein, our IHC protocol was titrated to discern normal and neoplastic cells with a particularly high TYMS expression and was therefore of low-to-intermediate sensitivity. The successful analysis of 15,361 cancers from 136 different tumor types still revealed detectable TYMS expression in a large fraction of cancers. Among the 136 tumor categories analyzed, there were 127 with TYMS positivity in at least one case, 71 with TYMS staining in at least 50% of cases, and 56 with at least one strongly positive case. The ranking order of our cancers according to their rate of positivity or strong positivity is a key finding of our study. It is worth noting that the selection of a more sensitive protocol would have resulted in higher numbers of positive cases, but—most likely—in a comparable ranking order. The impact of staining conditions and evaluation criteria on the outcome of IHC studies is highlighted by the variability of data in almost all tumor entities for which multiple IHC studies had evaluated TYMS expression (Figure 4). Examples of frequently analyzed cancer types include adenocarcinoma of the colon (20.0% to 100% positive in previous studies) [27,28], adenocarcinoma of the lung (20.0% to 100%) [29,30], breast carcinoma NST (21.0% to 100%) [18,31], and gastric adenocarcinoma (7.7% to 97.1%) [32,33].

The role of TYMS in cancer is most likely limited to thymine production, which is critically needed in the preparation for cell division [1,2,3]. The particularly high rate of TYMS positivity in high-grade lymphomas, sarcomas, sarcomatoid carcinomas, malignant melanoma, squamous cell carcinomas of various sites, and testicular germ cell tumors was expected and fits very well with the high proliferative activity known to frequently occur in these tumor types [39,40,41,42]. Accordingly, tumor entities known for a rather low proliferation rate, such as benign tumors, RCCs, low-grade prostatic adenocarcinomas, thyroid cancers, or neuroendocrine tumors, had particularly low rates and levels of TYMS positivity in our study. This is also in agreement with observations from earlier studies [43,44,45,46,47]. Also, in line with our findings in cancer, TYMS-positive normal tissues and cell types, such as germinal centers of lymphatic tissues, bone marrow, spermatocytes in the testis, crypt cells of the colorectum, and neck cells of the stomach epithelium, represented tissues or cell types known to have the highest proliferative activity [48,49,50,51,52].

Based on these observations, it appears likely that the significant associations between high TYMS expression levels and several adverse histopathological parameters in breast cancer, clear cell and papillary RCC, prostatic adenocarcinoma, colorectal adenocarcinoma, and squamous cell carcinoma may be driven by the role of TYMS expression in cell replication. Altogether, our findings in normal tissues and various cancer types are in line with a key role of TYMS expression for tumor cell proliferation. For several of these tumors, commercial RNA-based tests have been established that predict the natural disease course based on the measurement of multiple different genes [53]. All RNA-based tumor tests are compromised by the admixture of a variable quantity of non-neoplastic cells. Because of the rapid technological progress of multiplex fluorescence IHC enabling the simultaneous analysis of an increasing number of antibodies on one tissue slide, we anticipate that RNA-based prognosis tests will eventually be replaced by multicolor IHC tests. Whether TYMS could serve as a component of future IHC-based prognostic test panels remains a hypothesis that would need to be evaluated in dedicated prospective, treatment-stratified cohorts.

As rapidly proliferating tumors respond best to treatments with cytotoxic drugs, a favorable response of tumors with strong TYMS expression to these medications could be expected. In line with this assumption, patients with colorectal adenocarcinomas with high TYMS expression showed a favorable response rate to 5-FU therapy, and patients with breast cancers with high TYMS expression responded favorably to pemetrexed (antifolate) therapy in comparison to cancers with low-level TYMS expression [3,54]. However, one meta-analysis of 24 studies and two additional studies found that low-level TYMS expression was associated with favorable 5-FU therapy response in colorectal cancers [55,56,57]. One additional study showed an association of low TYMS expression and better response to pemetrexed in lung cancer [58]. A possible explanation could be that low TYMS expression levels might impair DNA repair and thus result in increased cancer cell apoptosis. This effect, combined with the DNA damage caused by 5-FU, might result in a higher rate of cancer cell death or therapy response [59].

It is of note that the assay used in this study was extensively validated by comparing our IHC findings in normal tissues with data obtained by another independent anti-TYMS antibody and RNA data derived from three different publicly accessible databases (https://www.proteinatlas.org/ENSG00000176890-TYMS/tissue, 12 July 2026) [60,61,62,63]. This approach follows the recommendations of the International Working Group for Antibody Validation [64]. The identification of cross-reactive antibody binding to proteins other than the target protein represents the most significant challenge for antibody validation, which is hard to achieve in cell line experiments. As the tissues from 76 different normal tissue categories (eight donors each) will contain most cell types of adult humans, a near-complete spectrum of human proteins with a broad spectrum of posttranslational modifications was evaluated for potential cross-reactivities in our study. The validity of our assay was supported by the preferential detection of TYMS staining in lymphatic and hematopoietic tissues (the tissues with the highest TYMS RNA expression) and the regular detection of TYMS staining in epithelial cells of the gastrointestinal tract, squamous epithelia, and the placenta, which were also shown to express TYMS RNA although at lower levels. That all cell types detected as TYMS-positive by HMV305 were also positive according to the independent second antibody TYMS/1884 represents further strong support for assay specificity. The additional staining of prostate epithelial cells by TYMS/1884 represents an antibody-specific cross-reactivity of TYMS/1884, which was unveiled by our comprehensive validation approach.

## 5. Conclusions

Our data demonstrate that TYMS is often overexpressed in a broad range of different cancer entities as compared to corresponding normal tissues. The observed associations of high TYMS expression with several adverse histopathological parameters across different tumor entities fit well with the known role of TYMS in cell proliferation. These findings may motivate future studies to assess whether TYMS IHC could inform patient selection for TYMS-inhibitor therapies.

## Figures and Tables

**Table 1:** TYMS immunostaining in tumors.

Tumor Category	Tumor Entity	TYMS Immunostaining					
		on TMA (*n*)	Analyzable (*n*)	Negative (%)	Weak (%)	Moderate (%)	Strong (%)
**Tumors of the skin**	Basal cell carcinoma of the skin	89	72	54.2	45.8	0.0	0.0
	Benign nevus	29	23	95.7	4.3	0.0	0.0
	Squamous cell carcinoma of the skin	145	114	40.4	51.8	5.3	2.6
	Malignant melanoma	65	54	9.3	46.3	38.9	5.6
	Malignant melanoma lymph node metastasis	86	78	29.5	53.8	14.1	2.6
	Merkel cell carcinoma	2	1	0.0	0.0	0.0	100.0
**Tumors of the head and neck**	Squamous cell carcinoma of the larynx	109	87	37.9	55.2	3.4	3.4
	Squamous cell carcinoma of the pharynx	60	59	30.5	50.8	15.3	3.4
	Oral squamous cell carcinoma (floor of the mouth)	130	117	24.8	54.7	18.8	1.7
	Pleomorphic adenoma of the parotid gland	50	33	97.0	3.0	0.0	0.0
	Warthin tumor of the parotid gland	49	37	91.9	8.1	0.0	0.0
	Basal cell adenoma of the salivary gland	15	11	63.6	36.4	0.0	0.0
**Tumors of the lung, pleura, and thymus**	Adenocarcinoma of the lung	196	163	62.6	31.9	3.7	1.8
	Squamous cell carcinoma of the lung	80	65	38.5	52.3	9.2	0.0
	Mesothelioma, epithelioid	40	37	59.5	35.1	5.4	0.0
	Mesothelioma, biphasic	29	26	53.8	42.3	3.8	0.0
	Thymoma	29	23	21.7	21.7	26.1	30.4
	Lung, neuroendocrine tumor (NET)	29	26	69.2	26.9	3.8	0.0
**Tumors of the female genital tract**	Squamous cell carcinoma of the vagina	78	54	33.3	55.6	9.3	1.9
	Squamous cell carcinoma of the vulva	157	136	35.3	51.5	11.8	1.5
	Squamous cell carcinoma of the cervix	136	122	22.1	52.5	23.0	2.5
	Adenocarcinoma of the cervix	23	23	21.7	52.2	26.1	0.0
	Endometrioid endometrial carcinoma	338	312	34.0	61.9	2.6	1.6
	Endometrial serous carcinoma	86	73	30.1	60.3	8.2	1.4
	Carcinosarcoma of the uterus	57	51	7.8	52.9	29.4	9.8
	Endometrial carcinoma, high grade, G3	13	13	0.0	61.5	30.8	7.7
	Endometrial clear cell carcinoma	9	8	50.0	50.0	0.0	0.0
	Endometrioid carcinoma of the ovary	130	115	34.8	59.1	6.1	0.0
	Serous carcinoma of the ovary	580	527	26.9	64.1	7.8	1.1
	Mucinous carcinoma of the ovary	101	84	60.7	36.9	1.2	1.2
	Clear cell carcinoma of the ovary	51	43	48.8	44.2	4.7	2.3
	Carcinosarcoma of the ovary	47	42	14.3	66.7	16.7	2.4
	Granulosa cell tumor of the ovary	44	43	44.2	46.5	9.3	0.0
	Leydig cell tumor of the ovary	4	4	100.0	0.0	0.0	0.0
	Sertoli cell tumor of the ovary	1	1	100.0	0.0	0.0	0.0
	Sertoli Leydig cell tumor of the ovary	3	3	66.7	33.3	0.0	0.0
	Steroid cell tumor of the ovary	3	3	100.0	0.0	0.0	0.0
	Brenner tumor	41	38	86.8	13.2	0.0	0.0
**Tumors of the breast**	Invasive breast carcinoma of no special type	1764	1643	48.9	46.2	4.2	0.7
	Lobular carcinoma of the breast	363	325	70.2	28.6	1.2	0.0
	Medullary carcinoma of the breast	34	32	15.6	50.0	28.1	6.3
	Tubular carcinoma of the breast	29	20	90.0	10.0	0.0	0.0
	Mucinous carcinoma of the breast	65	54	75.9	24.1	0.0	0.0
	Phyllodes tumor of the breast	50	47	87.2	12.8	0.0	0.0
**Tumors of the digestive system**	Adenomatous polyp, low-grade dysplasia	50	44	43.2	50.0	6.8	0.0
	Adenomatous polyp, high-grade dysplasia	50	43	23.3	69.8	7.0	0.0
	Adenocarcinoma of the colon	2483	2332	49.5	46.4	3.1	1.0
	Gastric adenocarcinoma, diffuse type	215	165	66.7	31.5	1.8	0.0
	Gastric adenocarcinoma, intestinal type	215	174	29.3	60.9	8.6	1.1
	Gastric adenocarcinoma, mixed type	62	55	40.0	56.4	3.6	0.0
	Adenocarcinoma of the esophagus	83	55	43.6	40.0	16.4	0.0
	Squamous cell carcinoma of the esophagus	76	48	35.4	52.1	10.4	2.1
	Squamous cell carcinoma of the anal canal	91	83	30.1	43.4	20.5	6.0
	Cholangiocarcinoma	58	52	61.5	30.8	5.8	1.9
	Gallbladder adenocarcinoma	51	47	51.1	46.8	2.1	0.0
	Gallbladder Klatskin tumor	42	34	70.6	23.5	5.9	0.0
	Hepatocellular carcinoma	312	306	64.7	32.4	2.6	0.3
	Ductal adenocarcinoma of the pancreas	659	565	67.1	30.1	2.7	0.2
	Pancreatic/Ampullary adenocarcinoma	98	86	50.0	46.5	3.5	0.0
	Acinar cell carcinoma of the pancreas	18	17	29.4	70.6	0.0	0.0
	Gastrointestinal stromal tumor (GIST)	62	58	79.3	13.8	6.9	0.0
	Appendix, neuroendocrine tumor (NET)	25	14	92.9	7.1	0.0	0.0
	Colorectal, neuroendocrine tumor (NET)	12	11	90.9	9.1	0.0	0.0
	Ileum, neuroendocrine tumor (NET)	53	47	85.1	12.8	2.1	0.0
	Pancreas, neuroendocrine tumor (NET)	101	97	74.2	20.6	5.2	0.0
	Colorectal, neuroendocrine carcinoma (NEC)	14	12	16.7	58.3	8.3	16.7
	Ileum, neuroendocrine carcinoma (NEC)	8	5	80.0	20.0	0.0	0.0
	Gallbladder, neuroendocrine carcinoma (NEC)	4	4	0.0	25.0	25.0	50.0
	Pancreas, neuroendocrine carcinoma (NEC)	14	12	58.3	33.3	8.3	0.0
**Tumors of the urinary system**	Non-invasive papillary urothelial carcinoma, pTa G2 low grade	87	71	22.5	74.6	2.8	0.0
	Non-invasive papillary urothelial carcinoma, pTa G2 high grade	80	62	9.7	74.2	12.9	3.2
	Non-invasive papillary urothelial carcinoma, pTa G3	126	101	23.8	54.5	14.9	6.9
	Urothelial carcinoma, pT2–4 G3	735	488	19.7	48.8	16.8	14.8
	Squamous cell carcinoma of the bladder	22	21	42.9	52.4	0.0	4.8
	Small-cell neuroendocrine carcinoma of the bladder	5	5	20.0	40.0	0.0	40.0
	Sarcomatoid urothelial carcinoma	25	21	33.3	47.6	9.5	9.5
	Urothelial carcinoma of the kidney pelvis	62	58	60.3	32.8	6.9	0.0
	Clear cell renal cell carcinoma	1287	1193	89.4	10.1	0.4	0.0
	Papillary renal cell carcinoma	368	329	82.7	16.7	0.6	0.0
	Clear cell (tubulo) papillary renal cell carcinoma	26	22	90.9	9.1	0.0	0.0
	Chromophobe renal cell carcinoma	170	153	96.7	3.3	0.0	0.0
	Oncocytoma of the kidney	257	217	93.5	6.0	0.5	0.0
**Tumors of the male genital organs**	Adenocarcinoma of the prostate, Gleason 3+3	83	74	97.3	2.7	0.0	0.0
	Adenocarcinoma of the prostate, Gleason 4+4	80	68	80.9	19.1	0.0	0.0
	Adenocarcinoma of the prostate, Gleason 5+5	85	75	76.0	24.0	0.0	0.0
	Adenocarcinoma of the prostate (recurrence)	258	170	68.2	28.2	2.9	0.6
	Small-cell neuroendocrine carcinoma of the prostate	2	1	100.0	0.0	0.0	0.0
	Seminoma	682	654	76.0	21.7	2.0	0.3
	Embryonal carcinoma of the testis	54	49	32.7	42.9	24.5	0.0
	Leydig cell tumor of the testis	31	31	80.6	19.4	0.0	0.0
	Sertoli cell tumor of the testis	2	2	50.0	0.0	0.0	50.0
	Sex cord stromal tumor of the testis	1	1	0.0	100.0	0.0	0.0
	Spermatocytic tumor of the testis	1	1	0.0	100.0	0.0	0.0
	Yolk sac tumor	53	43	32.6	55.8	11.6	0.0
	Teratoma	53	43	93.0	7.0	0.0	0.0
	Squamous cell carcinoma of the penis	92	86	33.7	43.0	22.1	1.2
**Tumors of endocrine organs**	Adenoma of the thyroid gland	113	105	100.0	0.0	0.0	0.0
	Papillary thyroid carcinoma	391	344	92.7	7.3	0.0	0.0
	Follicular thyroid carcinoma	154	116	92.2	7.8	0.0	0.0
	Medullary thyroid carcinoma	111	96	99.0	1.0	0.0	0.0
	Parathyroid gland adenoma	43	37	100.0	0.0	0.0	0.0
	Anaplastic thyroid carcinoma	45	39	48.7	46.2	5.1	0.0
	Adrenal cortical adenoma	48	47	91.5	2.1	6.4	0.0
	Adrenal cortical carcinoma	27	27	33.3	59.3	7.4	0.0
	Pheochromocytoma	51	50	86.0	8.0	6.0	0.0
**Tumors of hematopoietic and lymphoid tissues**	Hodgkin’s lymphoma	103	80	8.8	31.3	47.5	12.5
	Small lymphocytic lymphoma, B-cell type (B-SLL/B-CLL)	50	48	18.8	54.2	22.9	4.2
	Diffuse large B cell lymphoma (DLBCL)	113	113	3.5	28.3	37.2	31.0
	Follicular lymphoma	88	88	10.2	39.8	40.9	9.1
	T-cell non-Hodgkin’s lymphoma	25	24	4.2	33.3	29.2	33.3
	Mantle cell lymphoma	18	18	16.7	55.6	22.2	5.6
	Marginal zone lymphoma	16	15	20.0	33.3	40.0	6.7
	Diffuse large B-cell lymphoma (DLBCL) in the testis	16	16	6.3	25.0	31.3	37.5
	Burkitt lymphoma	5	4	0.0	0.0	25.0	75.0
**Tumors of soft tissue and bone**	Granular cell tumor	23	21	100.0	0.0	0.0	0.0
	Leiomyoma	50	48	93.8	6.3	0.0	0.0
	Leiomyosarcoma	94	86	66.3	23.3	5.8	4.7
	Liposarcoma	96	84	66.7	28.6	4.8	0.0
	Malignant peripheral nerve sheath tumor (MPNST)	15	13	46.2	23.1	30.8	0.0
	Myofibrosarcoma	26	26	46.2	53.8	0.0	0.0
	Angiosarcoma	42	36	25.0	47.2	16.7	11.1
	Angiomyolipoma	91	85	97.6	2.4	0.0	0.0
	Dermatofibrosarcoma protuberans	21	15	53.3	46.7	0.0	0.0
	Ganglioneuroma	14	14	100.0	0.0	0.0	0.0
	Kaposi sarcoma	8	5	20.0	60.0	20.0	0.0
	Neurofibroma	117	107	100.0	0.0	0.0	0.0
	Sarcoma, not otherwise specified (NOS)	74	70	41.4	47.1	8.6	2.9
	Paraganglioma	41	40	87.5	12.5	0.0	0.0
	Ewing sarcoma	23	17	41.2	35.3	17.6	5.9
	Rhabdomyosarcoma	7	7	0.0	57.1	28.6	14.3
	Schwannoma	122	117	99.1	0.9	0.0	0.0
	Synovial sarcoma	12	10	10.0	60.0	30.0	0.0
	Osteosarcoma	19	15	66.7	26.7	6.7	0.0
	Chondrosarcoma	15	10	50.0	30.0	20.0	0.0
	Rhabdoid tumor	5	4	0.0	25.0	25.0	50.0
	Solitary fibrous tumor	17	17	58.8	41.2	0.0	0.0

**Table 2:** TYMS immunostaining and tumor phenotype.

		*n*	TYMS Immunostaining				*p*
			Negative (%)	Weak (%)	Moderate (%)	Strong (%)	
Invasive breast carcinoma of no special type	pT1	775	52.5	43.6	3.5	0.4	0.0097
	pT2	627	43.9	49.9	5.3	1.0	
	pT3-4	123	39.0	55.3	4.9	0.8	
	G1	193	67.4	31.1	1.6	0.0	<0.0001
	G2	816	56.9	40.6	2.5	0.1	
	G3	561	28.9	61.7	7.7	1.8	
	pN0	922	54.1	41.4	3.9	0.5	0.9417
	pN+	673	52.8	42.8	4.0	0.5	
	pM0	210	59.0	37.6	2.4	1.0	0.1866
	pM1	112	46.4	49.1	3.6	0.9	
	HER2-negative	871	45.7	49.0	4.4	0.9	0.0673
	HER2-positive	126	37.3	60.3	1.6	0.8	
	ER-negative	209	33.0	56.0	8.1	2.9	<0.0001
	ER-positive	740	46.5	50.4	2.8	0.3	
	PR-negative	407	41.3	50.6	6.4	1.7	0.0002
	PR-positive	588	47.1	50.5	2.2	0.2	
	Non-triple-negative	780	45.1	51.8	2.8	0.3	<0.0001
	Triple-negative	140	32.1	52.9	10.7	4.3	
Clear cell renal cell carcinoma	ISUP 1	265	94.7	4.9	0.4	0.0	0.0022
	ISUP 2	399	90.2	9.5	0.3	0.0	
	ISUP 3	265	87.9	11.7	0.4	0.0	
	ISUP 4	73	76.7	21.9	1.4	0.0	
	Fuhrman 1	64	92.2	6.3	1.6	0.0	0.0030
	Fuhrman 2	677	91.4	8.4	0.1	0.0	
	Fuhrman 3	296	87.8	11.8	0.3	0.0	
	Fuhrman 4	88	76.1	22.7	1.1	0.0	
	Thoenes 1	349	94.0	5.7	0.3	0.0	0.0066
	Thoenes 2	490	90.0	9.8	0.2	0.0	
	Thoenes 3	95	81.1	17.9	1.1	0.0	
	UICC 1	321	91.3	8.1	0.6	0.0	0.0060
	UICC 2	38	89.5	10.5	0.0	0.0	
	UICC 3	93	87.1	11.8	1.1	0.0	
	UICC 4	71	84.5	15.5	0.0	0.0	
	pT1	668	89.5	10.2	0.3	0.0	0.7286
	pT2	135	91.1	8.9	0.0	0.0	
	pT3-4	327	88.4	11.0	0.6	0.0	
	pN0	172	90.7	9.3	0.0	0.0	0.0120
	pN+	24	70.8	29.2	0.0	0.0	
	pM0	109	91.7	8.3	0.0	0.0	0.0288
	pM+	91	81.3	18.7	0.0	0.0	
Papillary renal cell carcinoma	ISUP 1	34	91.2	5.9	2.9	0.0	0.0087
	ISUP 2	129	82.2	17.8	0.0	0.0	
	ISUP 3	81	81.5	17.3	1.2	0.0	
	ISUP 4	7	28.6	71.4	0.0	0.0	
	Fuhrman 1	1	100.0	0.0	0.0	0.0	0.0422
	Fuhrman 2	174	83.3	16.1	0.6	0.0	
	Fuhrman 3	83	84.3	14.5	1.2	0.0	
	Fuhrman 4	11	36.4	63.6	0.0	0.0	
	Thoenes 1	53	86.8	13.2	0.0	0.0	0.0422
	Thoenes 2	153	84.3	15.0	0.7	0.0	
	Thoenes 3	17	70.6	29.4	0.0	0.0	
	UICC 1	98	81.6	18.4	0.0	0.0	0.5462
	UICC 2	12	91.7	8.3	0.0	0.0	
	UICC 3	5	60.0	40.0	0.0	0.0	
	UICC 4	11	90.9	9.1	0.0	0.0	
	pT1	200	83.0	16.5	0.5	0.0	0.2373
	pT2	45	84.4	15.6	0.0	0.0	
	pT3-4	34	67.6	29.4	2.9	0.0	
	pN0	25	92.0	8.0	0.0	0.0	0.0045
	pN+	15	46.7	46.7	6.7	0.0	
	pM0	26	80.8	19.2	0.0	0.0	0.3509
	pM+	12	66.7	33.3	0.0	0.0	
Adenocarcinoma of the prostate	pT2	77	93.5	6.5	0.0	0.0	0.0149
	pT3	131	80.2	19.9	0.0	0.0	
	pT4	7	71.4	28.6	0.0	0.0	
	Gleason 3+3	74	97.3	2.7	0.0	0.0	<0.0001
	Gleason 4+4	68	80.9	19.1	0.0	0.0	
	Gleason 5+5	75	76.0	24.0	0.0	0.0	
Adenocarcinoma of the colon	pT1	86	36.0	57.0	4.7	2.3	<0.0001
	pT2	433	38.1	55.9	4.4	1.6	
	pT3	1255	49.6	46.6	3.1	0.7	
	pT4	435	58.2	38.9	2.3	0.7	
	pN0	1152	45.2	50.6	3.3	0.9	0.0050
	pN+	1051	52.7	43.2	3.1	1.0	
	V0	1593	45.8	49.6	3.5	1.1	0.0005
	V1	577	56.0	40.6	2.8	0.7	
	L0	711	43.9	52.5	2.7	1.0	0.0064
	L1	1472	51.0	44.6	3.5	1.0	
	Right side	455	38.0	52.3	6.8	2.9	<0.0001
	Left side	1226	52.0	45.8	1.7	0.6	
	MMR-proficient	1146	49.0	48.5	2.0	0.5	<0.0001
	MMR-deficient	84	16.7	61.9	9.5	11.9	
	RAS wildtype	468	49.4	46.4	2.4	1.9	0.0137
	RAS mutation	358	49.7	47.2	3.1	0.0	
	BRAF wildtype	123	47.2	51.2	1.6	0.0	<0.0001
	BRAF V600E mutation	22	13.6	59.1	13.6	13.6	
* Squamous cell carcinoma	pT1	258	25.6	53.9	18.2	2.3	0.2549
	pT2	263	33.1	51	14.8	1.1	
	pT3	133	33.1	51.1	12.8	3	
	pT4	126	38.1	46.8	11.9	3.2	
	pN0	296	32.1	50.7	14.9	2.4	0.1055
	pN+	304	30.9	53.6	13.2	2.3	
	G1	33	45.5	42.4	9.1	3	<0.0001
	G2	365	34.2	52.1	12.6	1.1	
	G3	243	20.6	52.7	22.2	4.5	

Abbreviation: pT: pathological tumor stage; G: grade; pN: pathological lymph node status; pM: pathological status of distant metastasis; MMR: mismatch repair protein; ISUP: International Society of Urological Pathology; UICC: Union for International Cancer Control. * Including squamous cell carcinomas of the oral cavity (*n* = 119), pharynx (*n* = 59), larynx (*n* = 82), esophagus (*n* = 36), lung (*n* = 59), cervix (*n* = 139), vagina (*n* = 54), vulva (*n* = 100), penis (*n* = 77), skin (*n* = 68), and the anal canal (*n* = 78).

## Data Availability

All data generated or analyzed during this study are included in this published article.

## Supplementary Materials

The following supporting information can be downloaded at https://zenodo.org/records/18667873, accessed on 13 July 2026, Figure S1: Assay validation by comparison of antibodies; Table S1: TYMS expression and cancer aggressiveness in endometrial and ovarian cancer.
